# The impact of physical activity on electronic media use among chinese adolescents and urban-rural differences

**DOI:** 10.1186/s12889-023-16103-x

**Published:** 2023-06-29

**Authors:** Shengchao Bai, Yutong Yin, Shengju Chen

**Affiliations:** 1 Department of Physical Education, Nanjing University of Science and Technology, Nanjing, 210094 China; 2 Department of Exercise Physiology, School of Physical Education, Liaoning Normal University, Dalian, 116029 China

**Keywords:** Adolescent, Physical exercise, Electronic media, Video games

## Abstract

**Objective:**

With the prevalence of electronic media use among Chinese adolescents and concerns regarding its potential negative consequences on their health and development, this study investigated the relationship between physical exercise and electronic media use. Utilizing data from the China Education Panel Survey, we examine the impact of physical activity on adolescents’ electronic media use.

**Methods:**

A simultaneous equation model, including two-stage least square and three-stage least square, was employed to estimate the impact of physical activity on electronic media use among adolescents. Self-control theory and media addiction theory were also used to analyze electronic media use in adolescents. Descriptive statistics were used to analyzed the data.

**Results:**

Chinese adolescents dedicated a substantial amount of time, averaging 2.95 hours per day, to electronic media activities. Increasing physical activity demonstrated an effective means to reduce electronic media use. Furthermore, the impact of physical activity on electronic media use exhibited urban-rural stratification, with family factors related to social class status primarily influencing electronic media use among urban students, while physical activity has a more pronounced influence among rural students.

**Conclusions:**

Promoting physical activity represents a compelling and effective strategy for curbing excessive electronic media use among Chinese adolescents, particularly in rural areas where physical activity has a stronger influence. In addition, controlling media entertainment and recreation time and enhancing social cohesion can help to weaken media interest. While changing the social class status of families in urban areas may be difficult in the short term, parents should be aware that physical exercise is an effective way to reduce their children’s use of electronic media. Our findings suggested that promoting physical activity may be a promising strategy for reducing excessive electronic media use among Chinese adolescents, particularly in rural areas where physical activity has a stronger influence.

According to the China Internet Development Statistics Report, as of June 2022, the rate of internet penetration has reached 74.4%, with students aged 10-19 constituting the largest group at 13.5% [1]. The rise of electronic media has led to its use by students in economically developed areas approaching that of school hours, essentially becoming a “second classroom” for students [2]. However, excessive use of electronic media can have negative impact on physical [3], psychological [4], and social well-being [5]. The Core Messages and Interpretation of Health Education for Chinese Adolescents (2018 edition) explicitly states that the use of e-products by adolescents, except for learning purposes, should not exceed one hour in order to prevent visual health damage [6]. Mobile phone addiction is particularly prevalent among 14-year-old adolescent, with a third of them leaving their phones on late at night, negatively impacting sleep quality [7]. Studies have found that as little as 30 min of mobile phone use can affect functional metabolism in the right frontal region [8], and irrational media behavior can lead to poor self-control and internet addiction, eventually resulting in structural brain damage [9]. Additionally, long-term exposure to virtual networks can cause adolescents to become isolated from real-life experiences, disrupt social support [10], and potentially contribute to psychological issues such as depression [11].

The current challenge is to determine effective ways to curtail electronic media use and promote positive use behaviors among adolescents. Previous studies have demonstrated that psychological guidance can moderately reduce media use [12, 13], and physical activity may serve as an important intervention by promoting rest and regulation of brain cells, as well as compassionate student exercise. While a few studies have examined the relationship between physical exercise and internet addiction [14–16], several limitations must be taken into account when interpreting the current findings: (1) The majority of subjects resided in single regions, or even in the same schools, neglecting the significant disparities across various regions in China. (2) The proliferation of smart devices has resulted in the integration of multiple electronic media which is having a considerable impact on adolescents. Nonetheless, previous experimental studies have not explored whether media substitution takes place subsequent to physical exercise intervention, leading to an insufficient conclusion. (3) Although the number of students who suffer from excessive mobile phone dependence or internet addiction is a relatively small proportion of adolescent media users, more studies have focused on using physical exercise as a means of treating these issues. To ensure the healthy development of Chinese adolescents, studies must include a significant number of adolescents to effectively control media use through physical exercise.

This study utilized a representative and sizable national dataset to examine the usage of electronic media among Chinese adolescents through descriptive statistics. The study also utilized a joint cube equation model to investigate the impact of physical exercise on media usage. Additionally, this study systematically analyzed the different factors that influence electronic media usage both at home and in school, and further explored the potential of physical exercise to reduce media usage among urban and rural adolescent groups.

## Design

### Data source and population

The data for this study was obtained from the China Education Panel Survey (CEPS), a systematic survey that was developed and implemented by Renmin University of China. The Survey adopted the Probability Proportionate to Size Sampling (PPS) sampling method, which involved multi-stage and multi-stratified sampling techniques. The participants were surveyed annually, and long-term follow-up surveys were conducted at 1, 3, 4, 7, 8, 17, and 27 years following their completion of junior high school [17–19].

### Variables

#### Explained variables

In present study, the duration of media exposure was utilized as the primary measure to assess electronic media use, which is widely recognized as a commonly utilized metric [20]. Media exposure duration was determined by converting time-grade questions into numerical values [19]. These values were then combined with the weekly frequency of media use to calculate the total time spent on electronic media. Exercise time was calculated by multiplying the exercise frequency (i.e., number of weekly exercise sessions) with the duration of each exercise session for every day of the week. This calculation yields the total weekly exercise time. To address the right-skewed distribution of the sample, the data was logarithmically transformed [21] to better approximate a normal distribution. The transformed data was then used for model analysis. To avoid excluding data with an exercise time of 0, a value of 0.01 was assigned before the conversion [22].

#### Explanatory variables

This study examined several personal factors that may affect media use and exercise among Chinese adolescents. First, accommodation type was classified as a categorical variable to assess whether living arrangements restrict student media use and exercise. Second, the type of registered permanent residence was recorded to determine whether differences in residential status impacted media use and exercise. Third, academic pressure was measured using the amount of time students spent on academic work from Monday to Friday. Participants rated their stress levels on a scale of one to six, and the average score was used as a measure of stress [21]. Fourth, the study assessed participants’ interests in mobile phone and video games. Finally, students’ fitness levels, sports interests, and participation in physical education tutoring classes were considered as potential influencing factors.

Family factors are important in understanding adolescents’ utilization of electronic media. Positive parenting interventions have shown effectiveness in reducing Internet addiction among adolescents who encounter harmful information [23]. Additionally, parental guidance and active involvement in their children’s media usage significantly influence their media literacy [24]. Therefore, family-related factors play a crucial role in shaping adolescents’ utilization of electronic media [25]. These factors manifest in various dimensions, including parental education as an indicator of the family’s social class status, as well as the quality of the parent-child relationship. Additionally, these variables also impact the amount of time children allocate to physical activities [19]. Parental educational level was categorized based on questionnaire responses, ranging from “no education” to “postgraduate and above” [21]. Occupational status was determined by the parents’ reported job type in the questionnaire [26, 27].

School factors, such as the school’s nature, quality, and availability of sports facilities, along with other relevant indicators that comprehensively reflect the school’s level, were chosen for analysis [18].

#### Control variables

Gender differences were observed in adolescents’ media use, with boys being more socially entertained by games than girls. Additionally, there were significant gender differences in the time spent playing sports. Therefore, gender was included as a variable in the analysis. Table 1 provides detailed descriptive statistics of the variables.

**Table 1:** Descriptive statistics for all variables

Name	Mean	SD	Minima	Maxima	Interpretation
Explained variables					
Media use time	20.632	16.385	0	81	Continuous variable, time spent per week
Physical exercise time	1.916	0.784	-2	3.845	Continuous variable, logarithm of time spent per week
Explanatory variables					
Accommodation	1.695	0.461	1	2	Category variable, yes = 1, no = 2
Type of registered permanent residence	1.466	0.499	1	2	Category variable, rural = 1, urban = 2
Academic pressures	2.286	0.824	1	6	Continuous variable, scale 1–6
Sports interest	0.356	0.479	0	1	Category variable, none = 0, have = 1
Mobile and video game interests	0.917	0.721	0	2	Class variables, none = 0, both = 2
Self-rated fitness	3.853	0.939	1	5	Continuous variable, scale 1–5
Physical education tutoring	0.076	0.264	0	1	Category variable, none = 0, have = 1
Family finances	2.811	0.611	1	5	Continuous variable, scale 1–5
Computers and networks	1.378	0.882	0	2	Class variables, none = 0, both = 2
Parental education	10.949	3.102	0	19	Continuous variable, years of education
Parental occupational status	60.806	12.385	46.550	85.160	Continuous variable reflecting career status
Parenthood	5.184	0.941	1	6	Continuous variable, scale 1-6
Parental media supervision	4.754	1.136	2	6	Continuous variable, scale 2-6
Parental Internet access	1.981	0.709	1	3	
Parents’ screen time	2.714	0.773	1	4	Continuous variable, scale 1-4
School sports facilities	4.921	1.209	3	9	Continuous variables, scale 3-9
School quality	3.950	0.862	1	5	Continuous variable, scale 1-5
Nature of school	1.081	0.273	1	2	Category variable, public = 1, private = 2
Control variables					
Gender	0.469	0.499	0	1	Category variable, F = 0, M = 1
Only child	1.560	0.496	1	2	Category variable, no = 1, yes = 2

*SD* means standard deviation

## Methods

### Model selection

As adolescents may also spend excessive time using electronic media, we began with preliminary Ordinary Least Squares (OLS) estimates in this study and found an inverse statistical effect (Table 2). This suggests not only a one-way cause-and-effect relationship between adolescents’ physical activity and electronic media use but also a possible mutual relationship. However, exercise time as an independent variable could become a factor in an alternative model, rendering the explanatory variable an uncontrolled random variable. The corresponding equation would also be a random variable, and OLS estimates alone could lead to the inherent problem of ignoring the variable, which may result in bias. Despite this, we used OLS as a reference framework owing to its simplicity of calculation and ability to provide preliminary analytical information [28]. Moreover, we established a simultaneous equations model of factors related to endogenous variables to reduce setup problems [29].

**Table 2:** Regression results of adolescents’ physical exercise and electronic media use time

	Electronic media use				Physical exercise			
	Model 1 ols	Model 2 2sls	Model 3 3sls	Model 4 3sls_iter	Model 1 ols	Model 2 2sls	Model 3 3sls	Model 4 3sls_iter
Physical exercise	-0.954^***^	-4.578^***^	-4.564^***^	-4.564^***^				
	(-0.215)	(-1.21)	(-1.209)	(-1.208)				
Media use					-0.002^***^	-0.003^**^	-0.003^**^	-0.003^**^
					(-0.001)	(-0.002)	(-0.002)	(-0.002)
Gender (male)	2.397^***^	2.593^***^	2.563^***^	2.562^***^	-0.015	-0.011	-0.011	-0.011
	(-0.342)	(-0.354)	(-0.353)	(-0.353)	(-0.012)	(-0.019)	(-0.019)	(-0.019)
Only child (Yes)	0.349	0.204	0.171	0.171	-0.051^**^	-0.051^***^	-0.050^**^	-0.050^**^
	(-0.384)	(-0.394)	(-0.393)	(-0.393)	(-0.020)	(-0.020)	(-0.020)	(-0.020)
Registered residence (city)	-1.344^***^	-1.196^***^	-1.198^***^	-1.198^***^	0.035^*^	0.035^*^	0.035^*^	0.035^*^
	(-0.403)	(-0.413)	(-0.413)	(-0.413)	(-0.021)	(-0.021)	(-0.021)	(-0.021)
Accommodation (No)	1.124^***^	1.123^***^	1.171^***^	1.171^***^	0.001	0.001	0.001	0.001
	(-0.412)	(-0.419)	(-0.418)	(-0.418)	(-0.021)	(-0.021)	(-0.021)	(-0.021)
Academic stress	1.064^***^	1.134^***^	1.151^***^	1.151^***^	0.0252^**^	0.0255^**^	0.0258^**^	0.0258^**^
	(-0.21)	(-0.215)	(-0.214)	(-0.214)	(-0.011)	(-0.011)	(-0.011)	(-0.011)
Parental education	-0.276^**^	-0.209^*^	-0.199^*^	-0.199^*^	0.017^***^	0.017^***^	0.017^***^	0.017^***^
	(-0.111)	(-0.115)	(-0.114)	(-0.114)	(-0.006)	(-0.006)	(-0.006)	(-0.006)
Parental occupational status	-0.053^***^	-0.054^***^	-0.052^***^	-0.052^***^	-0.001	-0.001	-0.001	-0.001
	(-0.0176)	(-0.0179)	(-0.0179)	(-0.0179)	(-0.001)	(-0.001)	(-0.001)	(-0.001)
Parenthood	-0.452^**^	-0.196	-0.187	-0.187	0.054^***^	0.052^***^	0.053^***^	0.053^***^
	(-0.182)	(-0.204)	(-0.203)	(-0.203)	(-0.001)	(-0.001)	(-0.001)	(-0.001)
Family finances	0.843^***^	0.900^***^	0.938^***^	0.938^***^	0.016	0.017	0.018	0.018
	(-0.294)	(-0.300)	(-0.299)	(-0.299)	(-0.015)	(-0.015)	(-0.015)	(-0.015)
School quality	-1.991^***^	-1.946^***^	-1.945^***^	-1.945^***^	0.005	0.002	0.003	0.003
	(-0.204)	(-0.208)	(-0.208)	(-0.208)	(-0.010)	(-0.011)	(-0.011)	(-0.011)
School nature	1.853^***^	1.799^***^	1.804^***^	1.804^***^	-0.007	-0.005	-0.005	-0.005
	(-0.675)	(-0.688)	(-0.687)	(-0.687)	(-0.034)	(-0.035)	(-0.035)	(-0.035)
Computer only	0.984	0.850	1.024	1.024				
	(-0.672)	(-0.685)	(-0.663)	(-0.663)				
Computer and network	2.670^***^	2.740^***^	2.629^***^	2.629^***^				
	(-0.477)	(-0.486)	(-0.471)	(-0.470)				
Interested in mobile phone or video games	5.225^***^	5.270^***^	5.156^***^	5.156^***^				
	(-0.386)	(-0.393)	(-0.382)	(-0.382)				
Interested in both mobile phone and video games	9.391^***^	9.271^***^	9.319^***^	9.320^***^				
	(-0.485)	(-0.495)	(-0.484)	(-0.484)				
Parental media supervision	-2.898^***^	-2.807^***^	-2.859^***^	-2.860^***^				
	-0.15)	(-0.156)	(-0.153)	(-0.153)				
Parents online (occasionally)	-0.174	-0.011	-0.250	-0.251				
	(-0.46)	(-0.471)	(-0.456)	(-0.456)				
Parents online (often)	-0.638	-0.438	-0.71	-0.711				
	(-0.564)	(-0.578)	(-0.560)	(-0.559)				
Parental screen time	2.485^***^	2.479^***^	2.439^***^	2.439^***^				
	(-0.215)	(-0.219)	(-0.212)	(-0.212)				
Health status					0.043^***^	0.043^***^	0.040^***^	0.040^***^
					(-0.009)	(-0.009)	(-0.009)	(-0.009)
Sports interests (yes)					0.214^***^	0.213^***^	0.219^***^	0.219^***^
					(-0.019)	(-0.0190)	(-0.018)	(-0.018)
Physical education tutoring class					0.190^***^	0.190^***^	0.176^***^	0.176^***^
					(-0.034)	(-0.034)	(-0.033)	(-0.033)
School sports facilities					0.032^***^	0.032^***^	0.035^***^	0.035^***^
					(-0.007)	(-0.007)	(-0.007)	(-0.007)
Constant	31.210^***^	35.400^***^	35.660^***^	35.660^***^	1.116^***^	1.141^***^	1.138^***^	1.138^***^
	(-1.912)	(-2.382)	(-2.367)	(-2.367)	(-0.093)	(-0.102)	(-0.102)	(-0.102)
N	7942	7942	7942	7942	7942	7942	7942	7942
R^2^	0.176	0.146	0.146	0.146	0.098	0.098	0.098	0.098

^***^P < 0.01, ^**^P < 0.05, ^*^P < 0.1; Corresponding standard error in bracket

### Model building

In this study, a joint cube equation model was used to analyze the relationship between time spent on electronic media and physical activity. Exercise duration was the main explanatory variable in the media use time equation, while time spent using electronic media was listed as the primary explanatory variable in the exercise time equation. The model also considered other relevant factors, such as household registration, residential status, academic pressure, parental education, parental occupational status, parent-child relationship, family finances, computer and internet status, interest in mobile phones and video games, parental media supervision, parental access to the internet and television, school quality and nature, physical fitness, sports interests, physical education tutoring classes, and school sports facilities. Gender and only child status were included as control variables.

Time spent on electronic media and physical activity as explanatory variables. Exercise duration was the main explanatory variable in the media use time equation, with household registration, residential status, academic pressure, parental education, parental occupational status, parent-child relationship, family finances, computer and Internet status, interest in mobile phones and video game, parental media supervision, parental access to the Internet and television, school quality and nature as the remaining explanatory variables, with gender and one child status as the control variables. In the exercise time equation, time spent using electronic media was listed as the primary explanatory variable, with household registration, accommodation, academic stress, parental education, parental occupational status, parent-child relationship, family finances, school quality and nature, physical fitness, sports interests, physical education tutoring classes, and school sports facilities as the remaining explanatory variables, and gender and only child status as the control variables. To reduce setup problems, a simultaneous equations model of factors related to endogenous variables was established. OLS was still used as a reference framework owing to its simplicity of calculation and ability to provide preliminary analytical information. To analyze the impact of adolescents’ physical activity on media use, STATA 15.0 software was utilized to process the model [30]. The model variable expression is simplified to:

1 $$Media\_t=a_0\;+\;a_1\;Exercise\_t\;+\sum_{k=16}^ka_kZ_k\;+\;\varepsilon_1$$

2 $$Exercise\_t\;=\beta_0\;+\;\beta_1\;Media\_t\;+\;\sum_{k=15}^k\beta_kZ_k\;+\;\varepsilon_2$$

The variable Media_t represents the amount of time an adolescent spends using electronic media, while Exercise_t represents the duration of their physical activity. The regression coefficient for Exercise_t is denoted by α_1_ in Eq. (1), while β_1_ represents the regression coefficient for Media_t in Eq. (2), where Exercise_t is used as the regressor and Media_t as the base regressor. The equations include constants α_0_ and β_0_, and z_k_ represents other variables, including explanatory and control variables at various levels. The corresponding coefficients for these variables are represented by α_k_ and β_k_, while the stochastic perturbations are denoted by ε_1_ and ε_2_.

### Model estimation

To satisfy the rank and order conditions of the simultaneous equation model, we employed both the two-stage least squares method (2SLS) and the three-stage least squares method (3SLS) due to the over-identification of the equations in this study [31]. The consideration of both the rank and order conditions of the simultaneous equation model is important when selecting estimation methods. Given that all equations in this study exhibit overidentification, the utilization of 2SLS and 3SLS methods is deemed appropriate [32]. Specifically, we employed the 3SLS method for estimation and the 2SLS method for auxiliary validation, aligning with the study’s requirements and variable settings. Additionally, we employed the ordinary least squares (OLS) method as a reference frame to test the robustness of the iterative 3SLS method.

## Results

### Analysis of adolescent media usage

The results showed that a majority of teens (58.2%) engaged in mobile phone gaming, which was higher compared to those who did not, and approximately one-third played video games, as presented in Table 3. Furthermore, an analysis of adolescents’ media usage time showed that boys spent significantly more time than girls (*P* < 0.01), as presented in Table 4. On average, Chinese teens spent 20.63 hours on activities such as watching television, using the Internet, and playing games per week, which translates to 2.95 hours per day. A single-sample t-test showed a significant increase of more than one hour (*P* < 0.01), which was more than three times higher than the national recommended usage time.

**Table 3:** Results of using time of mobile phones and video games (*N* = 9853)

	No	Yes
Proportion of people playing on mobile phones	4114(41.8%)	5739(58.2%)
Proportion of people playing video games	6557(66.5%)	3296(33.5%)

**Table 4:** Result description of media usage time

	Total (*N* = 9773)	Girls (*N* = 4642)	Boys (* N*= 5131)
Total time spent	20.632 ± 16.385	18.237 ± 14.526	22.795 ± 17.611^***^
Television watching	10.944 ± 9.369	10.188 ± 8.765	11.621 ± 9.827^***^
Online, gaming	9.678 ± 10.055	8.040 ± 8.646	11.165 ± 10.964^***^
Weekday time spent	10.450 ± 10.884	9.285 ± 9.799	11.499 ± 11.675^***^
Weekend time spent	10.182 ± 7.724	8.955 ± 6.804	11.296 ± 8.312^***^

Values are expressed as Mean ± SD, compared to girls ***P < 0.01

### Impacts of physical activity on adolescents’ electronic media usage time

In this study, a simultaneous equation model was used to test all explanatory and control variables, ensuring that the maximum variance inflation factor (VIF) was 2.2 and there were no significant tissues of multiple collinearity. The study estimated OLS, 2SLS, 3SLS, and iterative 3SLS models, corresponding to model 1, model 2, model 3, and model 4, respectively. The regression coefficient for time spent using electronic media and physical activity was significant, and the coefficient estimate in model 1 was lower than that in model 2 to 4, indicating a significant interaction between these two variables and suggesting that OLS with a single equation had a large estimation error. Models 1 to 4 showed that the time adolescents spent using electronic media was influenced by numerous factors, including the time spent in physical activity, which had a significant negative impact. These results indicate that increased physical activity time can significantly reduce the use of electronic media, all other factors being equal. Therefore, the data suggest that a large sample of sports interventions can be an effective strategy to reduce excessive electronic media use by adolescents.

The use of electronic media is influenced by various personal factors. Notably, gender plays a significant role in the time spent on electronic media, with girls spending less time compared to boys. Enrolled students from rural areas had significantly more electronic media time than their urban counterparts. Further, students not living in dormitories from Monday to Thursday spent more time on electronic media. The analysis showed a positive relationship between academic stress and time spent on media use to release stress. The most prominent factors influencing the use of electronic media were mobile phones and interest in video games. This factor ranked highest among all the ranking variables in the model, highlighting the dominant role of interest.

Parental education was positively associated with family cultural background, while parental occupation status was related to family hierarchy, and family finances were linked to the availability of better equipment. A more harmonious relationship with parents was associated with increased parent-child interaction, which in turn limited adolescent media use, with parents acting as media supervisors. Conversely, less effective restraint was achieved in relationships with less harmonious parents [33, 34].

Regarding school factors, students spent less time using media in higher-ranked schools and more time in private schools. These results indicate that differences in the management of schooling were critical in shaping adolescent media use (Table 2).

### Analysis of differences in adolescent electronic media use between rural and urban areas

Our findings indicated a significant impact of physical exercise time on both urban and rural students, with a stronger influence observed among rural students. A clear gender disparity in electronic media usage time was evident between the two groups of students, while academic stress had a positive association. Notably, no significant difference was found in electronic media usage time between male and female urban students, whereas in rural areas, where accommodation was not available, the difference was much more pronounced.

Parental education level, occupational status index, and family finances were significant family factors contributing to electronic media usage among urban students. In contrast, these factors were not evident among rural students.

School quality had a significant impact on students in both urban and rural areas. However, a clear distinction was observed between urban and private schools in terms of electronic media usage time, while no significant difference was found among rural schools (Table 5).

**Table 5:** The time difference of adolescents’ electronic media use between urban and rural areas

	Urban area			Rural area		
	Model 1 (ols)	Model 2 (2sls)	Model 3 (3sls)	Model 4 (ols)	Model 5 (2sls)	Model 6 (3sls)
Exercise time	-0.970^***^	-3.280^**^	-3.263**	-0.940^***^	-5.504^***^	-5.498^***^
	(-0.310)	(-1.603)	(-1.599)	(-0.298)	(-1.830)	(-1.826)
Gender (male)	2.764^***^	2.871^***^	2.869^***^	2.208^***^	2.527^***^	2.462^***^
	(-0.470)	(-0.479)	-0.477	(-0.496)	(-0.525)	(-0.522)
Only child (Yes)	-0.102	-0.271	-0.301	0.362	0.345	0.298
	(-0.528)	(-0.544)	-0.543	(-0.559)	(-0.575)	(-0.573)
Accommodation (No)	1.139	1.207	1.280	1.108**	1.056**	1.093**
	(-0.757)	(-0.764)	(-0.762)	(-0.502)	(-0.517)	(-0.515)
Academic pressure	0.825^***^	0.826^***^	0.835^***^	1.293^***^	1.469^***^	1.511^***^
	(-0.270)	(-0.272)	(-0.272)	(-0.323)	(-0.339)	(-0.338)
Parental education	-0.416^***^	-0.380^**^	-0.368^**^	0.0258	0.107	0.119
	(-0.148)	(-0.151)	(-0.151)	(-0.169)	(-0.177)	(-0.176)
Parental occupational status index	-0.066^***^	-0.068^***^	-0.067^***^	-0.014	-0.012	-0.010
	(-0.023)	(-0.023)	(-0.023)	(-0.028)	(-0.029)	(-0.0289)
Parenthood	-0.089	0.126	0.124	-0.735^***^	-0.516^*^	-0.488^*^
	(-0.255)	(-0.296)	(-0.295)	(-0.260)	(-0.281)	(-0.280)
Family finances	1.895^***^	1.984^***^	2.012^***^	0.086	0.110	0.158
	(-0.451)	(-0.458)	(-0.457)	(-0.392)	(-0.403)	(-0.401)
School quality	-1.958^***^	-1.975^***^	-1.963^***^	-1.783^***^	-1.692^***^	-1.692^***^
	(-0.303)	(-0.305)	(-0.304)	(-0.279)	(-0.289)	(-0.289)
School nature (Private)	5.569^***^	5.373^***^	5.395^***^	0.827	0.796	0.798
	(-1.427)	(-1.444)	(-1.440)	(-0.796)	(-0.818)	(-0.816)
Computer only	1.565	1.731	1.511	0.429	0.060	0.586
	(-1.092)	(-1.106)	(-1.090)	(-0.883)	(-0.919)	(-0.869)
Computer and network	2.788^***^	2.958^***^	2.736^***^	2.419^***^	2.472^***^	2.372^***^
	(-0.828)	(-0.842)	(-0.830)	(-0.602)	(-0.619)	(-0.587)
Interested in mobile phone or video games	5.539^***^	5.557^***^	5.544^***^	5.086^***^	5.164^***^	4.895^***^
	(-0.541)	(-0.545)	(-0.538)	(-0.546)	(-0.562)	(-0.536)
Interested in both mobile phone and video games	9.037^***^	9.058^***^	9.029^***^	9.657^***^	9.321^***^	9.488^***^
	(-0.651)	(-0.656)	(-0.649)	(-0.716)	(-0.748)	(-0.721)
Parental media supervision	-3.074^***^	-3.041^***^	-3.049^***^	-2.732^***^	-2.565^***^	-2.688^***^
	(-0.208)	(-0.210)	(-0.208)	(-0.216)	(-0.232)	(-0.223)
Parents online (occasionally)	-1.988^***^	-1.845^**^	-2.022^***^	0.446	0.657	0.316
	(-0.771)	(-0.783)	(-0.772)	(-0.585)	(-0.607)	(-0.574)
Parents online (often)	-3.045^***^	-2.843^***^	-3.079^***^	1.189	1.297	1.056
	(-0.850)	(-0.867)	(-0.855)	(-0.82)	(-0.843)	(-0.797)
Parental TV time	2.579^***^	2.542^***^	2.570^***^	2.250^***^	2.311^***^	2.147^***^
	(-0.286)	(-0.289)	(-0.285)	(-0.321)	(-0.330)	(-0.314)
Constant	28.99^***^	31.78^***^	31.72^***^	29.79^***^	34.78^***^	35.64^***^
	(-2.709)	(-3.323)	(-3.309)	(-2.811)	-3.499	(-3.462)
N	3754	3754	3754	4188	4188	4188
R^2^	0.206	0.195	0.195	0.157	0.11	0.109

^***^P < 0.01, ^**^P < 0.05, ^*^P < 0.1; Corresponding standard error in bracket

## Discussion

This study has found that Chinese adolescents tend to spend a considerable amount of time on electronic media, and that there is a negative correlation between physical exercise and electronic media use. Moreover, the time spent on electronic media was influenced by various factors such as personal, family, and school factors. Notably, there were significant disparities between urban and rural areas.

### The prevalence of electronic media use among chinese adolescents

Several studies have examined the current state of electronic media use among Chinese adolescents. For instance, a survey of college students revealed that the internet has become the most widely used medium, whereas traditional broadcasting media was the least used, indicating a shift in mainstream media forms. Research on mobile phone use has primarily focused on its negative impacts, such as mental health issues [35], academic performance [36], and physical health problems [8]. These examples highlight the increasing prevalence of electronic media-related issues among adolescents. Our study found that adolescents’ daily use of electronic media significantly exceeds the recommended national limit. This is partly due to mobile phone usage and video gaming becoming increasingly popular hobbies. According to the self-control theory, adolescents have limited self-control and are more susceptible to relying on electronic media. However, the widespread penetration of computers and networks in households means that media usage will only continue to expand. Reducing the number of electronic devices in households is an ineffective strategy that limits adolescents’ communication opportunities. Therefore, more effective methods for controlling media time usage need to be explored.

### The impact of physical activity on electronic media use time

Regular physical exercise has been found to have a significant inverse effect on the amount of time spent using electronic media. Mary et al. [37] reported a negative correlation between time spent on physical activity and video game usage in college students. Similarly, Motl et al. [38] found that increasing the frequency of recreational physical activity significantly reduced screen time and suggested increasing physical activity time among adolescents. Although there are notable differences between Chinese and foreign contexts in terms of schools, family backgrounds, and student circumstances, our results are consistent with these previous findings. Engaging in physical activity as a hobby can help divert adolescents from excessive dependence on mobile phones and computers when it is more appealing than electronic media. Moreover, besides regular school work, participating in extracurricular physical activities can also reduce the amount of time spent on electronic media, indicating a negative relationship between physical activity and electronic media use.

Exercise is an effective means to decrease media use and dependency. The Media Dependency Theory is a classical theory that explains the relationship between an individual’s dependence on a particular medium and their audience. This theory postulates that individuals become addicted to a medium when it is the only resource to meet their needs and desires [39]. This dependency on media can stem from factors such as self-esteem, leisure boredom, and the thrill of seeking out information. In contrast, exercise promotes feelings of excitement, pleasure, and self-worth. It helps individuals to cope with negative emotions and improves their self-esteem, confidence, and self-efficacy. Furthermore, physical exercise provides more opportunities for socialization, encourages face-to-face interaction, reduces reliance on virtual platforms, and reduces leisure boredom. Many sports activities offer athletes a strong sense of stimulation and satisfaction, fulfilling their specific psychological needs. Therefore, physical activity can serve as an alternative or resource for media use, ultimately leading to a reduction in media consumption among young people. However, physical activity can be physically exhausting, and adolescents may have limited stamina. After an intense workout, neurons and muscles require time to recover, affecting lifestyle habits and ultimately reducing electronic media use.

### Analysis of Media Use among Urban and Rural students

Adolescents’ electronic media use is influenced by various factors, and the mechanism differ between urban and rural student groups. Physical activity has significant spillover effects [21]. Considering personal, family, and school factors together in understanding the influence of physical activity on electronic media use can better reflect the multifaceted nature of exercise.

The amount of time adolescents spend on electronic media is influenced by a variety of factors, and the mechanisms vary between urban and rural student groups. Personal, family, and school factors all play a role in influencing electronic media use. Among the personal factors, gender, accommodation (rural), and interest in media use were found to be the main influences. Students living in urban areas have more leisure time than those living in rural areas. However, inappropriate use of electronic media can lead to excessive use, which could enhance social integration by fostering interest in sports and reducing the impact of social exclusion and media dependence [40]. Weakening interest in media use while increasing physical activity time can also benefit adolescents’ physical and mental health and provide additional advantages.

In addition to personal factors, factors such as computer network configuration, parental media supervision, parent-child relationship (rural areas), and parents’ TV time are all important in affecting children’s media use in the home environment. Parents can devote screen time to family activities, such as after-dinner walks, to reduce electronic media use and strengthen parent-child relationships through physical activity. Stronger parent-child relationships in rural student groups are associated with lower levels of media use, while good parent-child relationships significantly regulate the impact of parental supervision on adverse peer interactions [41], magnifying the role of regulation and multiplying the combined impact of physical activity.

Regarding family social class, parental education, parental occupational status index, and family financial status have been found to affect media use among urban adolescents, but not among rural students. This suggests that families with lower levels of parental education and occupational status and higher financial status in urban areas should pay more attention to their children’s media use. However, given the cumulative disadvantage of students in terms of educational outcomes [42], and the fact that family social class is a situation that cannot be changed in the short term, it is necessary to explore how to narrow the scope of problems caused by class differences. Increasing physical activity is a reasonable and effective means of media intervention and deserves parents’ attention.

Finally, the quality and nature of schools affect the amount of time students spend in urban environments. In urban areas, public and private schools have different educational philosophies and student management styles that have a broad and lasting impact on students. Lower quality and private schools in urban areas should focus more on physical activity, which can also reduce the time spent on student media and improve student health.

## Conclusions and recommendations

### Conclusions

In China, the prolonged use of electronic media among young people has raised concerns about its adverse effects on health. At the individual level, the amount of time adolescents spend using electronic media is influenced not only by the cultural capital of the family but also by school segregation. The use of electronic media by adolescents is characterized by internal differentiation, exhibiting urban-rural stratification with varying mechanisms of influence. For urban students, it is primarily influenced by family factors related to social class, whereas among rural students, it is more likely to be affected by physical activity levels.

### Recommendations

Cultivating sports interests among adolescents can lead to increased social integration and help reduce the influence of social exclusion on media addiction, thereby decreasing their interest in media use. Changing the social class of families in urban areas within a short period is challenging. Parents should be aware that physical exercise can effectively reduce their children’s use of electronic media. Rural families should take an active role in physical exercise and increase their level of supervision and control over their children’s media usage.

### Limitations

The study is limited by the lack of information on the intensity of physical activity in the sample, preventing the examination of the impact of exercise intensity on the outcomes due to data constraints.

## Data Availability

Publicly available datasets were analyzed in this study. This data can be found here: http://www.cnsda.org/index.php?r=projects/view&id=61662993 (assessed on 15 January 2022).
